# Demonstration and Significance of Independently Growing Mammary Carcinomas within the Cardiac Lumina in Mice, with Epithelial and Stromal Differentiation

**DOI:** 10.1038/bjc.1959.16

**Published:** 1959-03

**Authors:** Hemprova Ghosh

## Abstract

**Images:**


### DEMONSTRATION AND SIGNIFICANCE OF INDEPENDENTLY GROWING MAMMARY CARCINOMAS WITHIN THE CARDIAC LUMINA IN MICE, WITH EPITHELIAL AND STROMAL DIFFERENTIATION

HEMPROVA GHOSH

*From the Department of Surgical Pathology, Washington University School of Medicine and Barnard Free Skin and Cancer Hospital, Saint Louis, Missouri, U.S.A.*

Received for publication November 6, 1958

IN the course of the routine histological examination of the hearts of mice that died with mammary carcinoma, nodules of the tumor were found growing independently in the cardiac lumina, in a considerable number of cases. In the absence of any available earlier reference, it may be presumed that this is the first demonstration of the growth of carcinoma with formation of different histological patterns, consisting of both epithelial and stromal elements inside the cardiac lumina. The development of the stroma in a tumor growing in the medium of the circulating blood without direct contact of the host tissue contradicts the generally accepted theory that the stroma is supplied by the host tissue only and that for the maintenance and growth of the stroma, its direct continuity with the host tissue is essential.

The significance of this new finding with regard to the prognosis is also considered in this study.

### MATERIALS AND METHODS

The material presented here is based on the autopsy study of C3H strain non-pregnant female mice, obtained from the Roscoe B. Jackson Memorial Laboratory, Bar Harbor, Maine. The mice were allowed to die with mammary carcinoma, either spontaneous or transplanted. Tissues were fixed in 10 per cent neutral formalin and sectioned at 4 micron thickness. Hematoxylin and eosin stains were made routinely. Special stains for the detection of mucin, amyloid, iron, melanin, elastic tissue, reticulin, muscle, PAS positive material, etc., were used on some specimens. Single sections from the heart, other organs and the tumors were taken routinely. Serial sections were made of several hearts to verify the absence of attachment of the tumor embolus to the cardiac wall.

The analysis of metastatic spread, Table I, is based on the study of all autopsy cases in which the tumor, heart and lungs, together with other organs, were studied microscopically. This includes 21 mice with spontaneous and 684 with single transplanted carcinomas. These mice were used in our experimental chemotherapy program. Since no appreciable difference was noticed between treated and untreated groups, with respect to the histologic picture of the tumor,

TABLE I.—*Metastatic Spread of Mammary Carcinoma in Seven Hundred and Five C3H Female Mice*

| No. of autopsies | Generation of tumor | No. of hearts showing tumor growth in cardiac cavities | No. of hearts showing coronary occlusion by tumor growth | No. of lungs with metastases | No. of animals having tumors in the cardiac cavity and no lung metastasis | No. of livers with metastases | No. of kidneys with metastases | No. of spleens with metastases |
|---|---|---|---|---|---|---|---|---|
| 21 | 1st (i.e., spontaneous) | 3 (14%) | – | 6 (29%) | 1 (5%) | – | – | – |
| 370 | 2nd | 16 (4%) | 1 | 79 (21%) | 2 (0·5%) | 2 | 1 | 1 |
| 51 | 3rd | 11 (22%) | 1 | 21 (41%) | 6 (12%) | 1 | – | – |
| 129 | 4th | 23 (18%) | 2 | 40 (31%) | 10 (8% | 4 | 2 | – |
| 134 | 5th | 15 (12%) | 1 | 55 (41%) | 3 (2%) | – | – | – |
| Total 705 | | 68 (9·6%) | 5 (0·7%) | 201 (28·5%) | 22 (3·1%) | 7 (0·9%) | 3 (0·4%) | 1 (0·1%) |

both of these groups were included in this study. Among the transplanted carcinoma group there were 370 tumors of second generation, 51 of third generation, 129 of fourth generation and 134 of fifth generation. None of the hearts were opened during the autopsy examination. The whole heart was fixed without knowing whether or not a tumor embolus was present within.

Sections of the rarely enlarged nodes show metastatic tumor in less than 1 per cent of the total autopsy cases.

### OBSERVATIONS

The growth of the tumor inside the cardiac cavity, with the formation of various histologic patterns, is seen (Fig. 1 through 10). Epithelial and stromal characteristics of the adenocarcinoma of the breast are moderately well formed, as shown in solid alveolar (Fig. 1, 2), cystic papillary (Fig. 3, 4), acinous (Fig. 5), cystic (Fig. 6), and combined patterns (Fig. 9, 10). A tendency to capsule formation by the peripheral tumor cells is often seen (Fig. 1, 2, 6). Often the lining cell membrane appears as endothelial lining and mimics the appearance of tumor emboli lying in the lymphatic channels (Fig. 1, 2). Not infrequently formation of channels lined by a flattened tumor cell layer forming endothelium (Fig. 4) can be seen in these growing tumor emboli.

Cellular lysis is recognized in the tumor, resulting in the formation of cystic structures (Fig. 6) in a more or less solid tumor mass. Various sizes, ranging from small nodules to nodules large enough to fill right atrial (Fig. 9, 10) or right ventricular cavities (Fig. 7, 8) have been observed. Indentation and rupture of the cardiac wall, as a result of tumor growth inside the cardiac lumen is occasionally noticed (Fig. 8).

In sections of the heart, tumor nodules are occasionally noted in the lumen of the superior vena cava (Fig. 9) and in the pulmonary artery, while passing through the pulmonary valve (Fig. 8). Tumor nodules are seen only rarely in the cavity of the left atrium and left ventricle. The most common site is the right ventricular cavity ; next the right atrial cavity. The large size of the tumor emboli is a hindrance for its passage through the pulmonary valve and pulmonary artery. This may partially explain the absence of metastases to the lung in several cases in which tumor emboli are seen in the cardiac cavity (Table I). Occasionally deposition of fibrin from the circulating blood is seen incorporated with the tumor (Fig. 1). On two occasions, the tumor nodule was seen to have been fixed to the lip of the cardiac valve cusp, but there was no evidence of continuity between valve tissue and stroma of tumor embolus.

In all the autopsy cases of mice that died with mammary carcinoma, either transplanted or spontaneous, microscopic study showed that the most frequent site of metastasis is the lung. We have observed the frequent occlusion of the pulmonary arteries and their branches by the tumor emboli with or without involvement of the lung parenchyma. Attainment of a large size by the tumor emboli in the pulmonary arteries, without invasion of the arterial wall for a considerable distance, is often noticed (Fig. 12, 13). Infarction of the lung is frequently seen associated with vascular obstruction due to tumor emboli (Fig. 12, 13). This has been one of the major complications leading to the death of the animal. In less than 1 per cent of the cases the coronary vessels were blocked by tumor emboli (Fig. 11, Table I), without any significant changes in the surrounding muscles because of the vascular obstruction.

The tendency for the mammary carcinoma in mice to maintain the form of a discrete nodule, even when it reaches an immense size, is shown in Fig. 14. This tendency is not only noticed in the primary tumor and the tumor nodules growing inside the heart but can be seen in the microscopic picture of the tumor while it is invading nearby tissue (Fig. 15, 16).

### DISCUSSION

The presence of metastatic tumor nodules inside the heart, in various situations, shows their course along the route of venous blood circulation, i.e., from superior vena cava to right atrium, right ventricle, pulmonary artery and then lungs. For all practical purposes, it may be assumed that the metastatic spread to the heart is carried by blood vessels, since we have noticed many blood vascular channels showing tumor in their lumina and since the lymph node involvement by this tumor is very rare, as mentioned before and noticed by other workers. It occurred in less than 1 per cent of our mice. The frequent finding of occlusion of the large and main branches of the pulmonary arteries by tumor nodules is presumably the result of impaction of growing tumor emboli, which have already grown to be a large size in their sojourn in the cardiac cavity. Occlusion of the large pulmonary vessels leading to infarction of the lungs appears to be an immediate cause of death in a considerable number of animals.

It is also suggested from this study that in some cases the gain in size of the tumor nodules in the cardiac lumen stopped their further progression along the path of circulation. This probably explains the absence of metastases in the lungs in a few cases, while the cardiac cavity is filled with a large tumor nodule.

The marked tendency for this tumor to form a well-outlined nodule, as shown in Fig. 14, 15 and 16, probably plays an important role in the pattern of growth of tumor emboli inside the cardiac lumen and in the pulmonary arteries and may be related to the very infrequent metastasis to other organs.

Complete indifference of the mammary carcinoma to invasion of the heart muscle and endocardium is noteworthy. Indentation of the cardiac wall and, finally, rupture because of the tumor growth still without invasion by the tumor, demonstrates the incompatibility of the cardiac muscle and endocardium with this tumor growth. Sugarbaker (1952) demonstrated by inoculating different tumors into the central arterial system of the rabbit that, in addition to establishment of its own metastatic pattern, a definite predilection exists peculiar to each tumor for the site of metastasis. His conclusion is that factors inherent in the type of tumor used and influences exerted by the organs of the host both contribute to produce these effects. Lucke, Breedis, Woo, Berwick and Nowell (1952) also show the differential growth of metastases in liver and lung in their experiments with Rabbit V2 carcinoma.

The growth and stromal differentiation of the tumor inside the cardiac cavity, independent of the host tissue, as shown in this study, is in contradiction to the generally accepted theory that the stroma is contributed by the host tissue only (Bashford, Murray and Cramer 1905*a*, 1905*b*; Haaland 1908). This theory emphasizes that, unlike the parenchyma, the stroma is devoid of the power of independent growth and proliferation and that the stroma of a tumor, either spontaneous or transplanted, introduced at inoculation undergoes degeneration and is replaced by supporting structures derived entirely from the tissues of the new host. It is important to recall that the detailed observations made by Loeb (1901) did not agree with this generally accepted theory that the stroma is derived from the host tissue only.

We have studied the various dynamic processes in the mechanism of formation of different tumor patterns and have observed the stromal differentiation by the neoplastic epithelial cells in mammary carcinoma of the mouse in both spontaneous and transplanted tumors (data to be published).

The growth of the tumor in the medium of the circulating blood, as presented here, probably represents an ideal condition of *in vivo* culture of the tumor for its epithelial and stromal differentiation. It is conceivable, as newer tissue inter- and intra-relationships are discovered, that continual or repeated supply of extracts or preparations from different tissues and organs could maintain the tumors under study more or less in their original structural and functional states entirely *in vitro*.

### SUMMARY

Ten percent of seven hundred and five C3H strain female mice bearing either transplanted or spontaneous mammary carcinoma showed development of tumors with stromal and epithelial differentiation inside the cardiac lumen. The finding that these tumors had no direct connection with the host tissue, except the circulating blood, disputes the generally accepted theory that the stroma develops from the host tissue only.

The increase in size of the tumour growing inside the cardiac lumen has its implications for the impediment of cardiac function by obstruction, and also with regard to the pattern of metastatic spread, as shown and discussed in this study.

This investigation was supported by the Copher Research Fund.

Grateful acknowledgement is made to Mrs. Dixie McGregor; Miss Fern Probstmeyer and Miss Tutter Levinsen for technical assistance, Mr. Cramer Lewis,

Department of Illustration, Washington University School of Medicine, for photomicrographs, and Mrs. Rose Valle for secretarial help.

### REFERENCES

BASHFORD, E. F., MURRAY, J. A. AND CRAMER, W.—(1905*a*) *Sci. Rep. Imp. Cancer Res. Fnd.*, **2**, 24.—(1905*b*) *Ibid.*, **2**, 30

HAALAND, M.—(1908) *Ibid.*, **3**, 175.

LOEB, L.—(1901) *J. med. Res.*, **6**, 28.

LUCKE, B., BREEDIS, C., WOO, Z. P., BERWICK, L. AND NOWELL, P.—(1952) *Cancer Res.*, **12**, 734.

SUGARBAKER, E. D.—(1952) *Cancer*, **5**, 606.

### EXPLANATION OF PLATES.

### PLATE I

FIG. 1.—Multiple tumor nodules are lying inside the right ventricular cavity, Note the presence of delicate cell membranes (A) loosely encapsulating the tumor tissue, better shown in the following figure. Deposition of fibrin can be seen on the surface of two nodules. Tumor emboli growing inside the pulmonary arteries are shown in Fig. 13 in a section of the lung. 5th generation tumor. × 40.

FIG. 2.—This is a higher magnification of the preceding photomicrograph (A), showing the formation of the capsule by the peripheral tumor cells. 5th generation tumor. × 380.

FIG. 3.—Metastatic tumor forming a multiloculated cystic papillary pattern inside the right ventricular cavity of a mouse bearing mammary carcinoma. No connection of this tumor with the cardiac wall is seen in serial section ; the cardiac wall is free of tumor tissue. Note the delicate pattern of the tumor with well-differentiated stroma. A magnified view of the tumor tissue (A) is shown in the following figure. 2nd generation tumor. × 45.

FIG. 4.—This is a magnified view of a portion of the preceding photomicrograph (A). A narrow channel (pointed by an arrow) lined with endothelium-like flattened cells is shown here. 2nd generation tumor. × 340

### PLATE II

FIG. 5.—Tumor emboli in the right ventricle forming an acinar pattern are exhibited here. No lung metastasis was seen in this animal. 2nd generation tumor. × 54.

FIG. 6.—Two isolated tumor nodules growing inside the right ventricular cavity are shown here. Note the formation of the capsule. Necrosis and dissolution of necrotic tumor tissue are seen in the formation of cystic cavities. No lung metastasis was seen in this animal. 4th generation tumor. × 38.

FIG. 7.—The right ventricular cavity is distended with a tumor growth. Prominent necrosis and hyaline degeneration can be seen in the tumor. No lung metastasis was seen in this case. Spontaneous tumor. × 22.

FIG. 8.—This photomicrograph demonstrates rupture of the right ventricular wall, due to the growth of the tumor inside the right ventricular cavity. Note the indentation marks over the inter-ventricular septum made by the growing tumor. A tumor nodule on its way to the pulmonary artery is shown on the left. The tumor nodule is seen lying against a pulmonary valve cusp. Lung metastasis was present in this case. 4th generation tumor. × 13.

### PLATE III

FIG. 9.—The right atrial cavity is distended with the growing carcinoma, which shows combination tumor patterns. The two cusps of the right atrio-ventricular valve are shown below the main tumor growth. The atrial wall is thickened. A tumor embolus can be seen in the inter-atrial septum distending a coronary vessel. The mitral valve cusps can be seen in left center. A tumor nodule on the right upper corner distends the superior vena cava. Lung metastasis was present in this case. 3rd generation tumor. × 27.

FIG. 10.—The growth of a delicate metastatic tumor, showing a combination of different histological carcinomatous patterns can be seen in the right atrium, mostly surrounded by blood. Tricuspid valve cusps point downward. The left atrial cavity of this heart, which is not shown here, also contains a small tumor nodule. 2nd generation tumor. × 40.

FIG. 11.—In the inter-ventricular septum of a heart, a coronary vessel is filled with solid carcinoma cells. No other tumor metastasis was seen in the heart. Section of the lungs showed pulmonary metastasis. 5th generation tumor. × 180.

FIG. 12.—This photomicrograph shows a portion of a lung at its hilum. The tumor growing inside the pulmonary artery is seen here. The arterial wall in most part is not attached to the tumor, which has a rounded margin. At higher magnification, some of the peripheral tumor cells were seen to take the form of flattened endothelium. The rest of the lung shows hemorrhagic infarction. 2nd generation tumor. × 24.

### PLATE IV

FIG. 13.—This photomicrograph shows blockage of the pulmonary artery by the growth of the tumor embolus and infarction of the lung. Figure 1 is also from the same mouse. 5th generation tumor. × 33.

FIG. 14.—A C3H strain mouse with spontaneous breast carcinoma. Note the well-outlined tumor forming a large discrete nodule. Spontaneous tumor. × $\frac{4}{5}$.

FIG. 15.—This photomicrograph exhibits invasion of the chest wall muscle by the mammary carcinoma as a block—a frequent feature of this type growth. 1st generation tumor. × 70.

FIG. 16.—Invasion of the chest wall musculature by the tumor is shown here at a lower magnification. Note the pattern of invasion ; the invading tumor nodules have well-outlined boundaries. 4th generation tumor. × 26.

Ghosh.

Ghosh.

Ghosh.


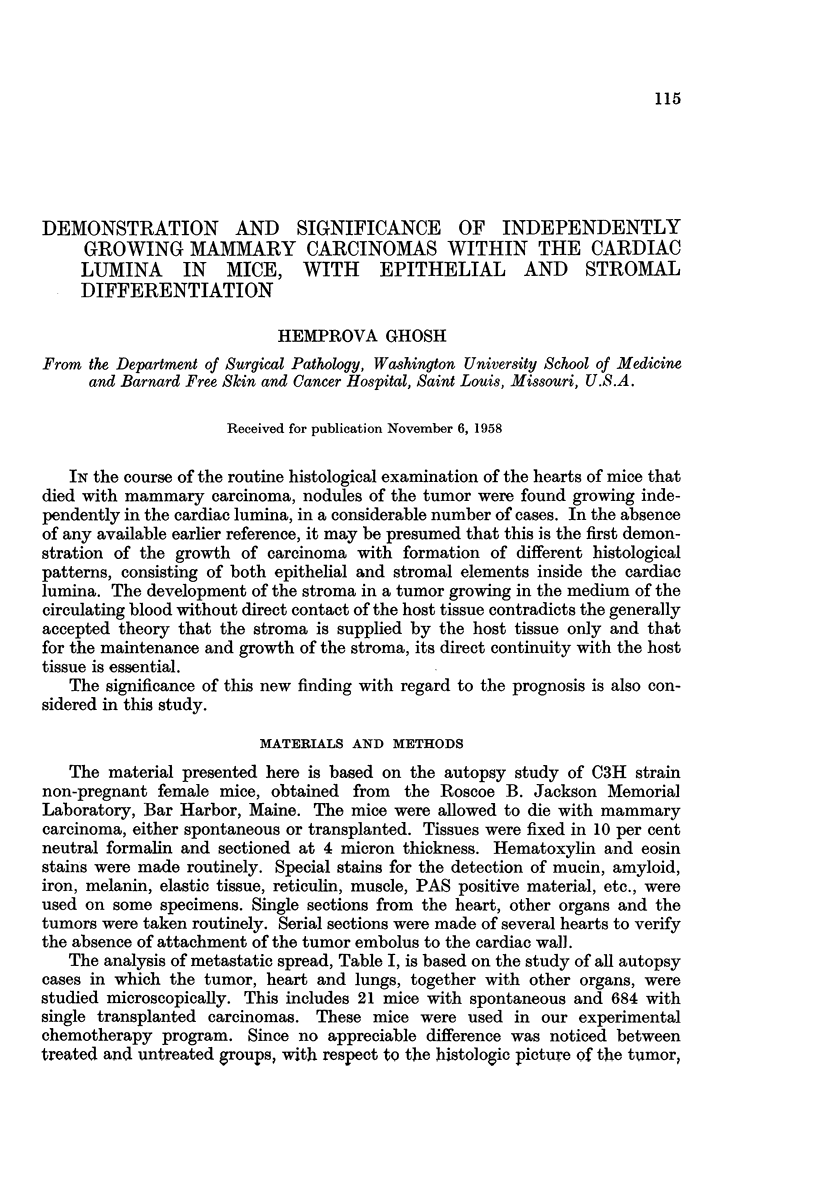

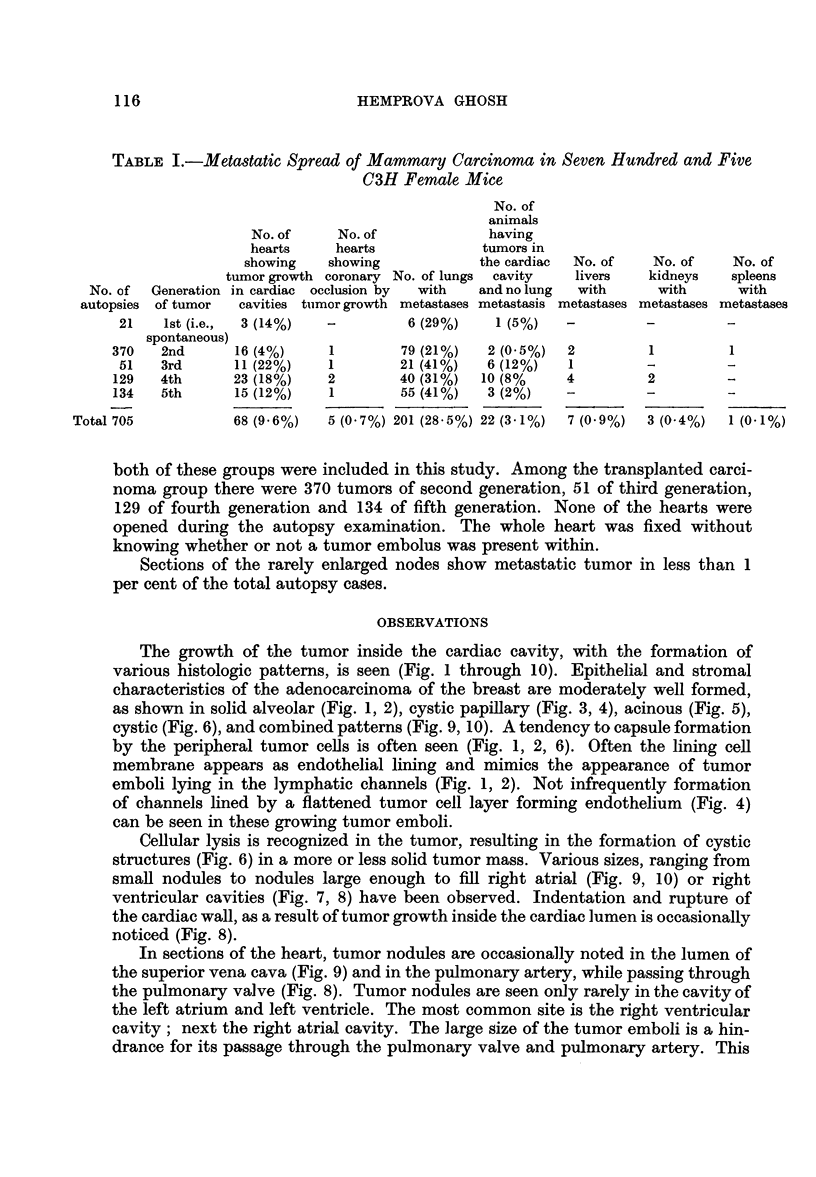

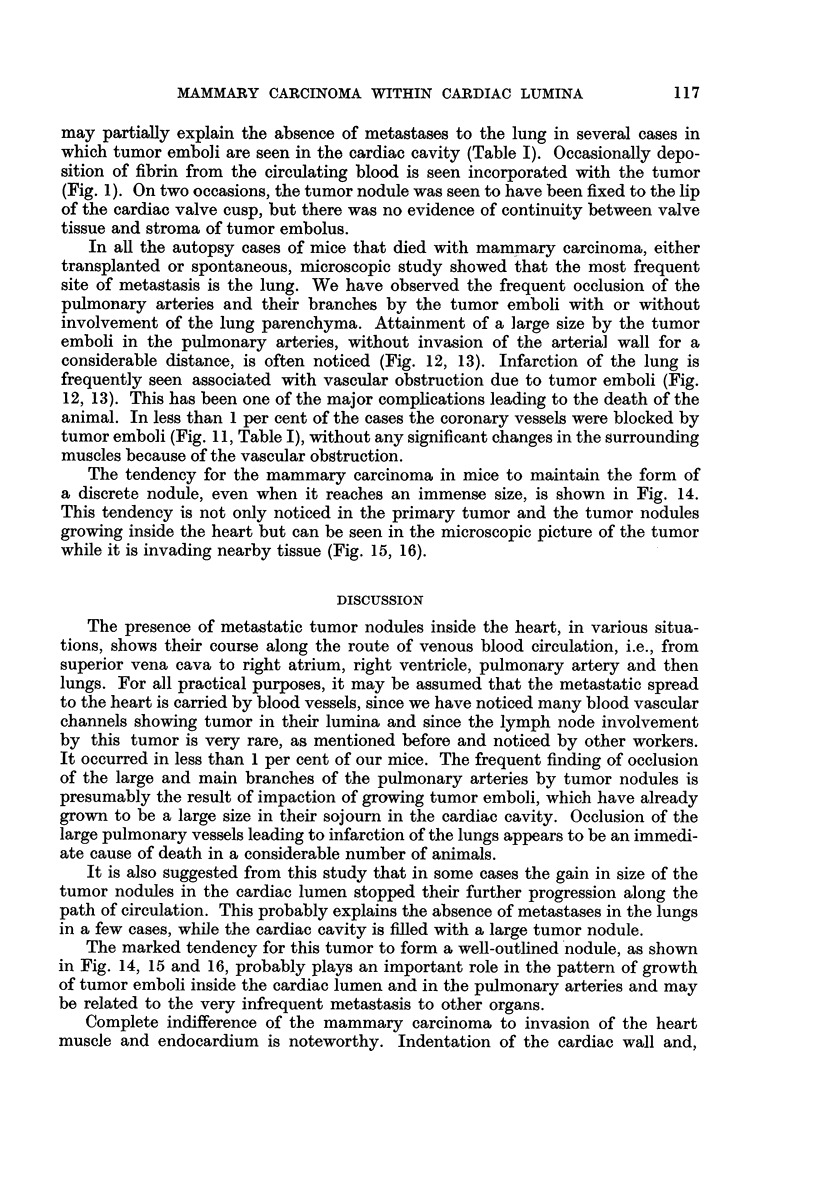

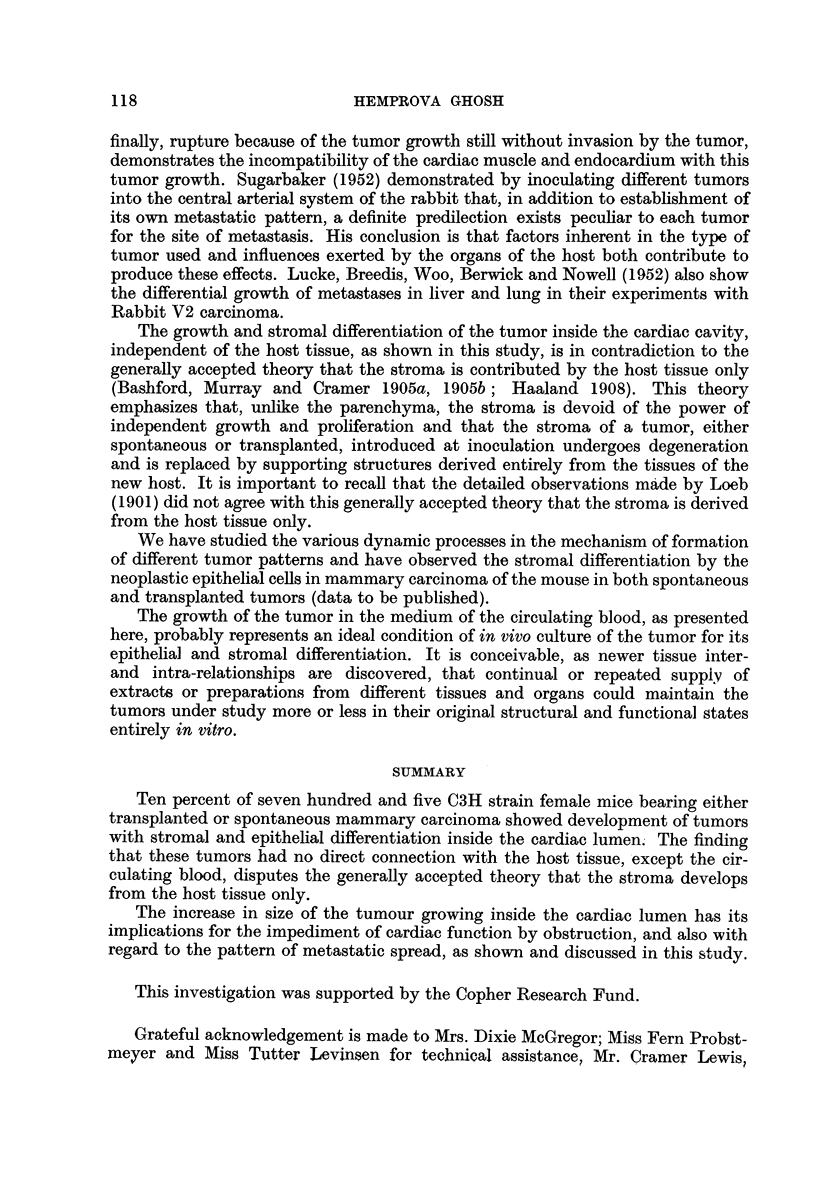

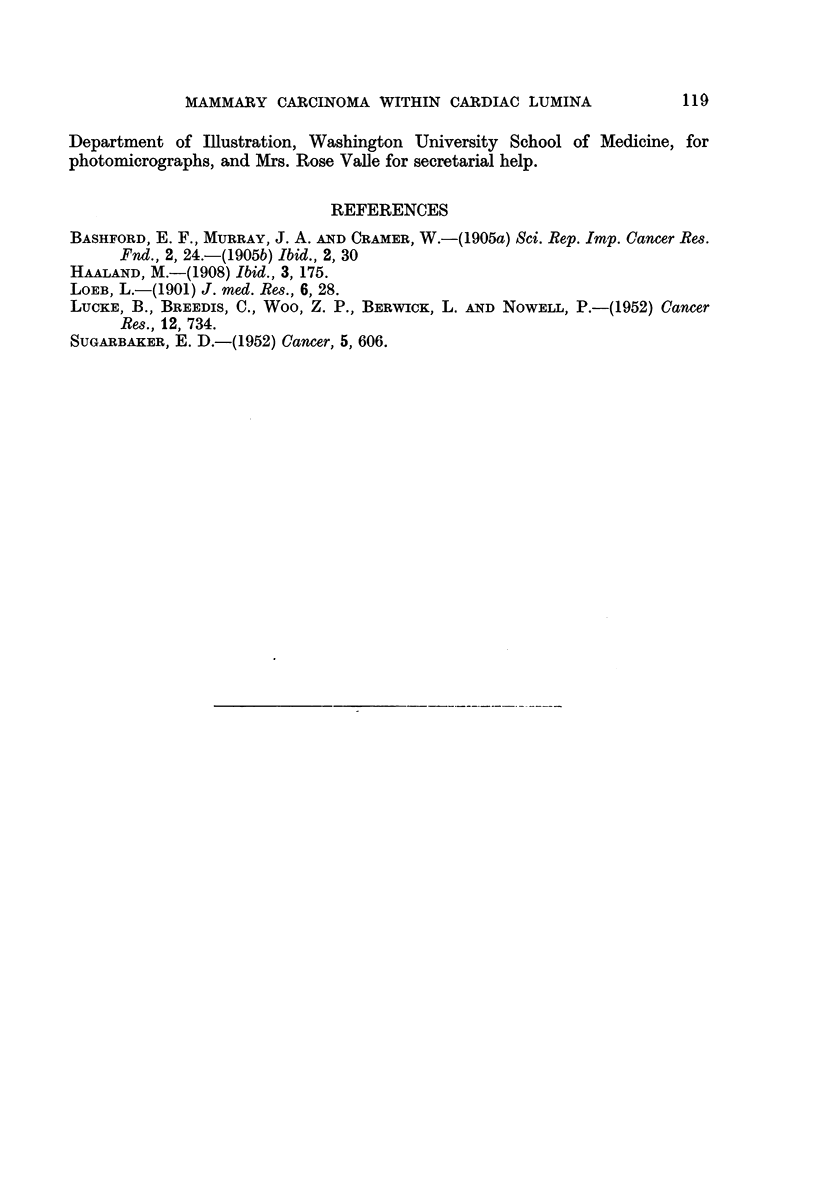

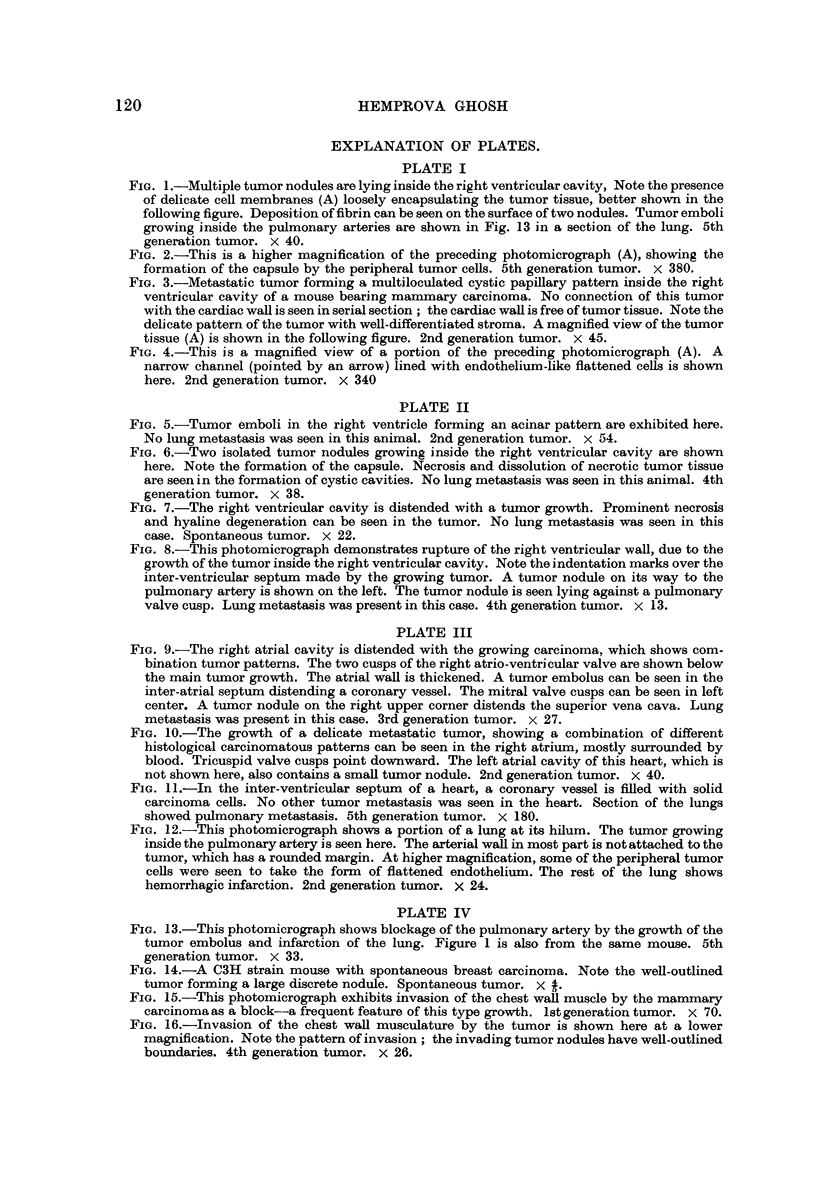

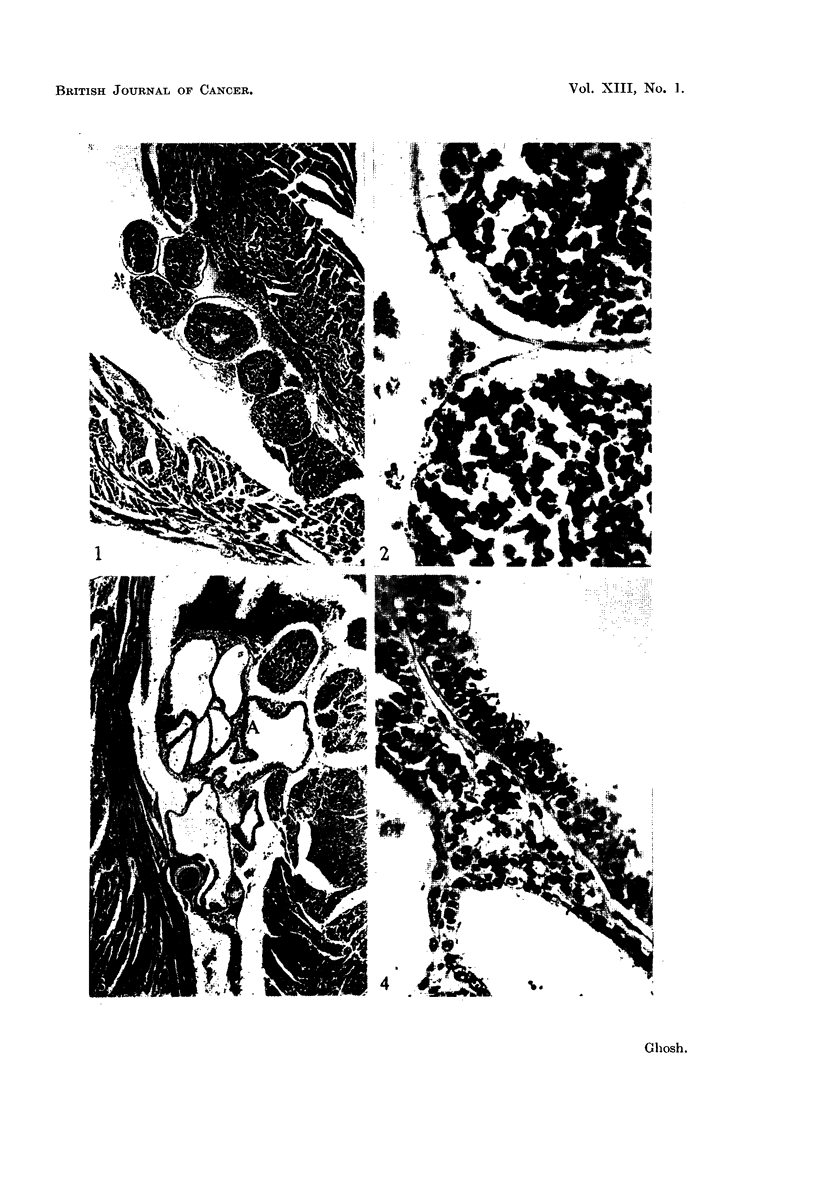

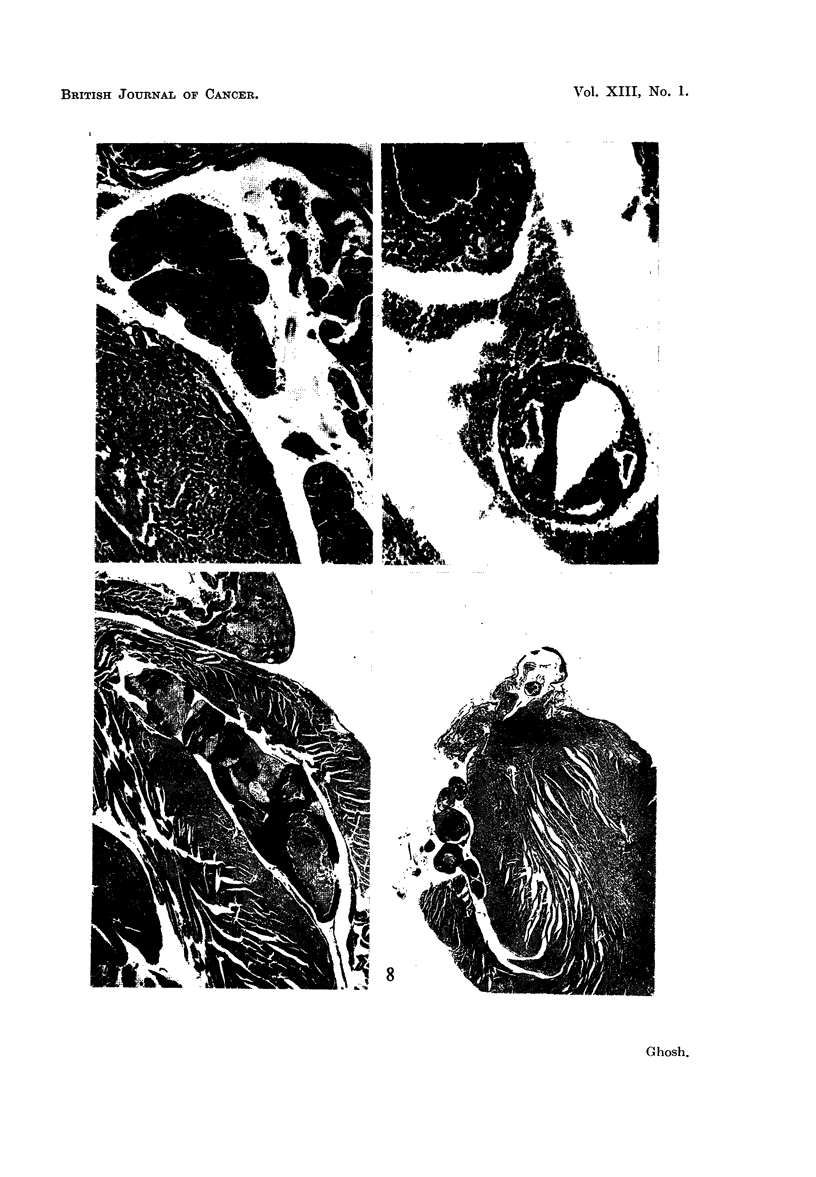

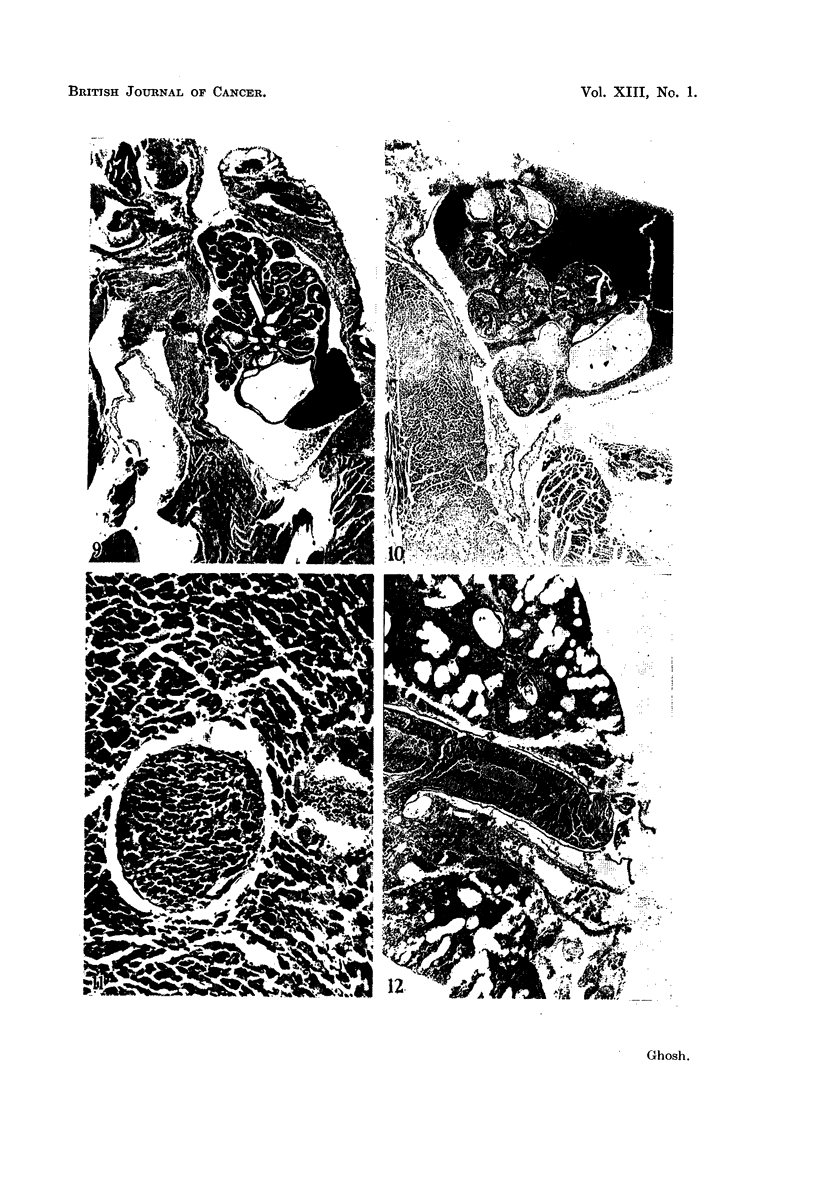

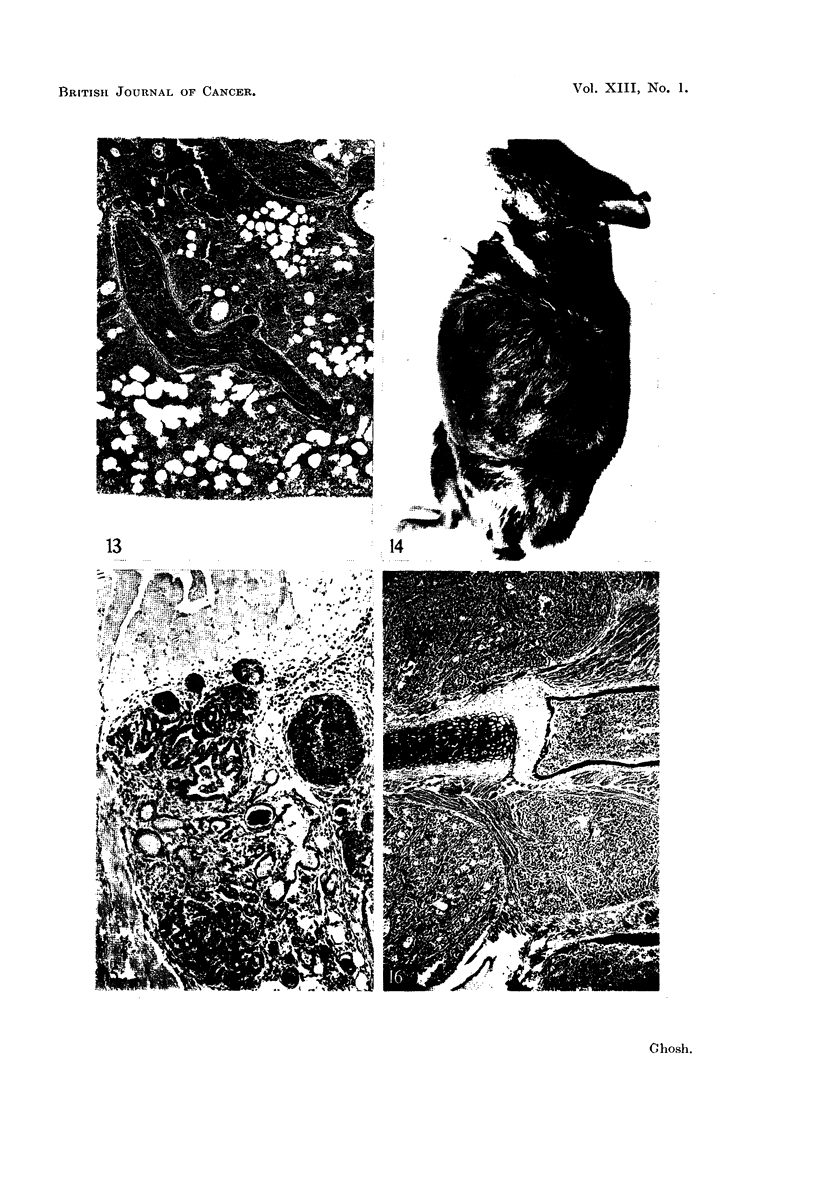


## References

[OCR_00275] HEWER T. F., MEEK E. S. (1958). Intestinal carcinoma in mice following injection of herring-sperm deoxyribonucleic acid.. Nature.

[OCR_00254] LUCKE B., BREEDIS C., WOO Z. P., BERWICK L., NOWELL P. (1952). Differential growth of metastatic tumors in liver and lung; experiments with rabbit V2 carcinoma.. Cancer Res.

[OCR_00276] NAORA H. (1955). Microspectrophotometry of cell nucleus stained by feulgen reaction. I. Microspectrophotometric apparatus without Schwarzschild-Villiger effect.. Exp Cell Res.

[OCR_00278] RIS H., MIRSKY A. E. (1949). Quantitative cytochemical determination of desoxyribonucleic acid with the Feulgen nucleal reaction.. J Gen Physiol.

[OCR_00256] SUGARBAKER E. D. (1952). The organ selectivity of experimentally induced metastases in rats.. Cancer.

